# High prevalence of common mental disorders in mothers of children with congenital Zika syndrome at the end of early childhood

**DOI:** 10.17179/excli2024-7591

**Published:** 2024-08-19

**Authors:** Carolina Santos Souza Tavares, Raquel Souza Marques, Paulo Ricardo Martins-Filho

**Affiliations:** 1 Investigative Pathology Laboratory, Federal University of Sergipe, Sergipe, Brazil; 2 Graduate Program in Health Sciences, Federal University of Sergipe, Sergipe, Brazil; 3 Graduate Program in Dentistry, Federal University of Sergipe, Sergipe, Brazil

## ⁯⁯⁯⁯

The Zika virus epidemic that affected South America, Central America, and the Caribbean from 2015 onwards profoundly impacted public health and raised important questions about its long-term consequences. Congenital Zika Syndrome (CZS) is a well-documented consequence of Zika virus infection during pregnancy, characterized by severe neurodevelopmental abnormalities in affected infants, including intracranial calcification, ventriculomegaly, reduced cerebral volume, microcephaly, auditory and visual deficits, and neuromuscular disorders (van der Linden et al., 2016[8]; Moore et al., 2017[5]). Given the challenges of this condition, many mothers of CZS-affected children experience significant alterations in their occupational roles, negatively impacting their quality of life and mental health (Carla da Silva Reis et al., 2020[2]; Morris et al., 2022[6]; Araújo et al., 2023[1]). While early postpartum effects on mothers have been studied (Dos Santos Oliveira et al., 2017[3]), long-term impacts remain under-investigated. Here, we investigated the prevalence of common mental disorders (CMDs) in mothers of 5-6 year old children with CZS. 

This cross-sectional study was conducted in the state of Sergipe, located in Northeast Brazil, a region which is recognized as the poorest in the country. In our study, we analyzed a group of 49 mothers whose children were diagnosed with CZS, representing approximately 42 % of all mothers with CZS-affected children in Sergipe. CZS diagnoses were confirmed at birth by medical teams according to the 2016 Brazilian Ministry of Health criteria, which require a head circumference at birth below two standard deviations for the gestational age and sex, alongside confirmed maternal or neonatal Zika virus exposure. Where viral detection via maternal or neonatal serology was unfeasible, alternative infectious and non-infectious causes of reduced head circumference were extensively examined and excluded. 

Mothers underwent face-to-face interviews in a private setting. These interviews were conducted by a researcher (CSST), who was not only previously trained but also boasted over 10 years of experience in the nursing field, with a focus on mental health. To assess CMDs - which are characterized by symptoms such as anxiety, depression, and unexplained somatic complaints - we employed the Self-Reporting Questionnaire (SRQ-20), a widely recognized tool in mental health research. In this questionnaire, each item is scored as 0 or 1, indicating the absence or presence of a symptom in the past month, respectively. A total score ranging from 0 to 20 is calculated, with a score of 8 or higher suggesting the presence of CMDs (Harding et al., 1980[4]). CMD prevalence and 95 % confidence intervals (CI) were estimated using the Clopper-Pearson method. 

The median age of the children in our study was 6 years, with an interquartile range (IQR) of 5 to 6 years, and all displayed functional delays as assessed by the Pediatric Evaluation of Disability Inventory Computer Adaptive Test (Pedi-CAT). The majority of the mothers had a median age of 30 years (IQR=25-36), with 77.6 % in a marriage or stable relationship and 83.7 % not participating in paid work. Regarding family income, nearly 94 % of the families earned between 1 to 3 Brazilian minimum wages monthly. As for the mental health assessment using the SRQ-20, scores ranged from 0 to 16, with a median of 6 (IQR=3-10). Twenty mothers scored 8 or higher, indicating a high prevalence of CMD symptoms at 41 % (95 % CI 27-56). 

The alarming prevalence of 41 % for CMDs in mothers of children with CZS, even years after the child's birth, underscores the multifaceted psychological and socioeconomic repercussions associated with the condition. In addition to economic vulnerability and reduced occupational opportunities, these mothers face a significant caregiving burden. The demands and clinical severity of CZS require constant, intensive care, which can lead to chronic stress, emotional exhaustion, and increased risk of mental health disorders. This caregiving stress, compounded with the families' financial instability, exacerbates the mothers' risk of CMDs. Our previous findings (Santos et al., 2021[7]) align with this, highlighting that diminished personal competence in mothers significantly correlates with psychological distress and a decline in social quality of life. 

These findings underscore the necessity for health policies that provide comprehensive long-term support, addressing the mental health needs of these mothers, mitigating the caregiving burden, and enhancing resilience and coping strategies. Such policies should ensure a holistic response to the complex challenges posed by CZS, ultimately improving the overall well-being of affected families.

## Declaration

### Ethics

The study was approved by the Research Ethics Committee of Federal University of Sergipe (CAAE 51713821.3.0000.5546).

### Funding

None. 

### Conflicts of interest

None. 

### Acknowledgments

PRMF is a research productivity fellow at the National Council for Scientific and Technological Development (CNPq), Brazil. CSST and RSM thank the Coordination of Higher Education and Graduate Training (CAPES) for his scholarship (Finance Code 001).

### Authors contributions

CSST: Conceptualization, methodology, investigation, formal analysis, writing - original draft, writing - review & editing; RSM: Conceptualization, methodology, investigation, writing - review & editing; PRMF: Conceptualization, project administration, methodology, formal analysis, writing - original draft, writing - review & editing. 
